# Identifying named entities from PubMed® for enriching semantic categories

**DOI:** 10.1186/s12859-015-0487-2

**Published:** 2015-02-21

**Authors:** Sun Kim, Zhiyong Lu, W John Wilbur

**Affiliations:** National Center for Biotechnology Information, National Library of Medicine, National Institutes of Health, Bethesda, 20894 MD USA

**Keywords:** Semantic term extraction, Natural language processing, Machine learning

## Abstract

**Background:**

Controlled vocabularies such as the Unified Medical Language System (UMLS®) and Medical Subject Headings (MeSH®) are widely used for biomedical natural language processing (NLP) tasks. However, the standard terminology in such collections suffers from low usage in biomedical literature, e.g. only 13% of UMLS terms appear in MEDLINE®.

**Results:**

We here propose an efficient and effective method for extracting noun phrases for biomedical semantic categories. The proposed approach utilizes simple linguistic patterns to select candidate noun phrases based on headwords, and a machine learning classifier is used to filter out noisy phrases. For experiments, three NLP rules were tested and manually evaluated by three annotators. Our approaches showed over 93% precision on average for the headwords, “gene”, “protein”, “disease”, “cell” and “cells”.

**Conclusions:**

Although biomedical terms in knowledge-rich resources may define semantic categories, variations of the controlled terms in literature are still difficult to identify. The method proposed here is an effort to narrow the gap between controlled vocabularies and the entities used in text. Our extraction method cannot completely eliminate manual evaluation, however a simple and automated solution with high precision performance provides a convenient way for enriching semantic categories by incorporating terms obtained from the literature.

**Electronic supplementary material:**

The online version of this article (doi:10.1186/s12859-015-0487-2) contains supplementary material, which is available to authorized users.

## Background

Due to the rapid growth of biomedical literature, machine learning and natural language processing (NLP) techniques have gained in popularity for (semi-)automatically extracting useful information [1]. A fundamental step in biomedical language processing is to identify terms representing entities, e.g. genes, organisms, diseases and chemicals [2]. High-level information extraction such as event extraction and biological network discovery comes next after necessary terms are correctly identified [3].

A term represents a particular concept an author intends to discuss, and the goal of term identification is to recognize the term and capture its underlying meaning [4]. Hence, term identification and concept extraction are often used interchangeably [5,6]. Approaches for term identification fall into three categories [1,4,7]: dictionary-based, rule-based and statistical-based. Dictionary-based approaches utilize existing terminological resources in order to identify term occurrences in text [4]. Since simple dictionary look-up has limited effectiveness, lexical knowledge of target terminologies is often employed as well [8-10]. Rule-based approaches find terms by building rules that describe naming structures for a certain concept [11-13]. These methods accurately identify known patterns, however manual rule construction is costly and time-consuming. The rules designed for a concept normally cannot be applied to other concepts. Statistical (or machine learning) approaches rely on word distribution for discriminating term and non-term features [14-16]. The key to successfully train a statistical model is annotated corpora [17-20], and the limited availability of such gold-standard sets is one of the main difficulties. It is also challenging to choose a set of discriminating features in statistical approaches.

Although there are a plethora of works addressing the term identification problem, most of them focus on only one or a few biological concepts [5]. This is because rule- and statistical-based approaches usually depend on special naming conventions or patterns specific to terms of interest. Many biomedical applications, however, require recognizing numerous classes of terms rather than recognizing only a few term classes [5,21]. The use of existing terminologies through dictionary-based or hybrid approaches has advantages in this regard. The Unified Medical Language System (UMLS) [22], for example, comprises more than two million concepts obtained from over 100 resources. While UMLS was not primarily created for natural language processing and text mining, it has been shown that UMLS can be successfully applied to certain biomedical and clinical problems [23-26].

A major pitfall of using controlled vocabularies such as UMLS and Medical Subject Headings (MeSH) for text mining is their low usage in biomedical literature [21]. UMLS aims at representing biological concepts and relationships and MeSH is for indexing articles and books in the life sciences. Thus, it is understandable to have little overlap between standard terminologies and terms used in written communication. McCray et al. [27] found 10% of the UMLS terms appeared in a set of 439,741 MEDLINE abstracts. Using flexible string matching techniques, Srinivasan et al. [28] reported that 34% of the UMLS terms were found in titles and abstracts in MEDLINE. A recent study [29] also showed only 518,835 UMLS terms (13%) appeared in MEDLINE. SemCat [30,31] is a database containing semantically categorized entities for genomics. More than 10 knowledge resources including UMLS, GENIA [18], Entrez Gene [32] and UniProt [33] were used and it contains over 10 million entries. We tested how many SemCat terms are consistent with PubMed text in the following sense. A SemCat term was represented by all contiguous word bigrams appearing in it. A SemCat term was rated consistent with PubMed text if all its bigrams appeared in PubMed text. Our analysis revealed that 41% of SemCat terms were compatible with PubMed abstract text.

The low overlap between UMLS and PubMed text has led to a few efforts for enriching controlled vocabularies. Mostly, it has been done by either filtering UMLS terms [21,27,29,34] or reclassifying UMLS concepts [35,36] for NLP problems. Bodenreider et al. [37], however, suggested an idea of using adjectival modifiers and demodified terms to extend the UMLS Metathesaurus. In this approach, terms were extracted from MEDLINE with 83% accuracy. Here, we address the same task, i.e. to extend a controlled vocabulary by adding new terms found in biomedical articles. The method we propose is based on how sentences are constructed in English, and does not require complicated NLP techniques. If a headword represents a unique concept, noun phrases that employ the headword become candidate terms. Since our goal is to extend existing terminologies, we apply three linguistic patterns to find new terms related to these candidates. The first pattern gives conditions which allow one to remove the headword. The second and third patterns find terms that are defined by headwords. After these procedures, a support vector machine (SVM) classifier is used to filter out noisy phrases. For experiments, SemCat [31] was used for training the classifier, and the extracted terms were manually evaluated by three annotators. The headwords used for the experiments were “gene”, “protein”, “disease”, “cell” and “cells”. The results showed over 93% precision on average for the three extraction patterns.

The paper is organized as follows. In the next section, we describe our approach that uses linguistic patterns and machine learning classifiers for extending a controlled vocabulary. This is followed by results and discussion for the experiments performed on SemCat and PubMed abstracts. Conclusions are drawn in the last section.

## Methods

Our approach consists of three steps to identify semantic terms from PubMed. The first step is to obtain headwords uniquely corresponding to concepts. The concept of a phrase is mostly determined by the headword. Hence, this procedure guarantees that we always examine the same concept phrases. The next step is to extract candidate terms using linguistic patterns. This process either removes the headword or finds neighboring terms that are semantically linked to the headword. However, the terms extracted from the linguistic patterns may be noisy, thus a SVM classifier is applied to eliminate irrelevant terms in the last step.

### Ambiguity of headwords

In a named entity, a word to the right is more likely to be the headword or the word determining the nature of the entity than a word to the left [38]. Therefore, if a headword represents a unique concept, the named entity with the headword most likely conveys the same concept.

Table 1 shows our analysis on the headwords for the gene/protein category in SemCat. For each term, either the last word or if a preposition is present, the last word before the preposition was considered as a headword. We first chose the headwords that appeared more than 20 times in this SemCat category. Second, the SemCat terms with these headwords were filtered by SVM classifiers. These SVM classifiers were built in the same way described in the following subsections. A reviewer judged the ambiguity of the selected headwords. In the table, “gene” and “protein” are always used for gene/protein names. “regulator” and “antigen” are often used for gene/protein names, but it is difficult to determine the correct category without considering the context. Some headwords such as “region” and “domain” are labeled as “No”. This is due to incorrect terms appearing in SemCat. Most of these cases represent terms where a protein name is followed by the headword, “region” or “domain”, placing these terms in a different category. Our analysis of these headwords may in some cases be debatable. However, it suggests that there are many unambiguous headwords.

**Table 1:** **Ambiguity of headwords for gene/protein names in SemCat**

**Gene/protein**	**Ambiguity**	**Headwords**
Yes	No	gene, protein, kinase, receptor, transporter, pseudogene, enzyme, peptide, polypeptide, glycoprotein, lipoprotein, symporter, antiporter, collagen, polyprotein, cotransporter, crystallin, lectin, globin, tubulin, oncogene, phosphoprotein, ferredoxin, opsin, antibody, porin, flavoprotein, homeobox, actin, adhesin, isoenzyme, integrin, lysozyme, chaperonin, globulin, ribonucleoprotein, immunoglobulin, isozyme, cadherin, transcript, myosin, apoprotein, cyclin, autoantigen, hemoglobin, spectrin, cytochrome, flagellin, tropomyosin, kinesin, adaptin, keratin, peroxiredoxin, pilin, chemokine, casein, catenin, ferritin, enkephalin, histone, giardin, interferon, albumin, trypsin, glutaredoxin, metallothionein, cyclophilin, proteolipid, mucin, vasopressin, proteoglycan
Ambiguous	Low	-ase (i.e. terms ending in “ase”), regulator, antigen, isoform, inhibitor, repressor, hormone, toxin, ras, carrier, suppressor, ligand, translocator, phosphate, thioredoxin, neurotoxin
	High	Greek letters (e.g. alpha, beta,...), Roman numerals, short strings (e.g. psi, orf, ib,...), precursor, subunit, homolog, chain, factor, component, family, product, channel, activator, system, variant, chaperone, superfamily, molecule, pump, exchanger, element, sequence, resistance, construct, allergen, exporter, transducer, sensor, finger, modulator, effector, antiterminator, fusion, defective, antagonist, locus, wing, acid, receiver, para, cofactor, spot, tail, pigment, class, coma, exon, interactor, coactivator
	Rarely used	content, percentage, gain, frame, length, ratio, response, yield, defect, fiber, resistant
No	No	region, domain, complex, form, fragment, binding, weight, transport, member, cell, containing, fluid, related, associated, syndrome, putative, biosynthesis, repeat, activity, segment, preparation, smear, subfamily, dependent, terminus, substrate, determinant, site, level, motif, specific, subtype, mrna, dna, synthesis, fibroblast, cdna, cluster, assembly, membrane, mutant, transmembrane, virus, terminal, group, hybrid, flip, urine, function, number, periplasmic, yield, rich, plasmid, rate, metabolism, fold

For each term, either the last word or the word before a preposition was considered as a headword. The uniqueness and the ambiguity for being a gene/protein name were judged by an annotator.

For our study, we chose “gene”, “protein”, “disease”, “cell” and “cells” for unique concept headwords. “gene”, “protein” and “disease” are frequently used as singular in PubMed, whereas the plural is more common for “cell”. Hence, we examined “cells” in addition to the singular, “cell”.

### Linguistic patterns for term extraction

The phrases that have unique concept headwords do not require further investigation as semantic categories are already determined by the headwords. For example, any phrases ending with “protein” always represent the concept, “protein” in PubMed. One way to identify other phrases with the same concept is to find phrases that share the same semantic property. Thus, we propose three linguistic patterns that find candidate phrases within or near unique concept phrases.

#### Linguistic Pattern 1

**Figure 1:**
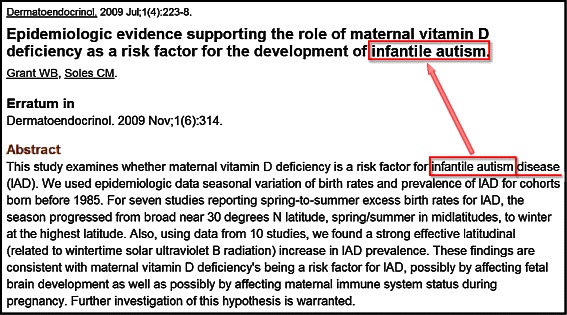
**An example for Linguistic Pattern 1.** This pattern evaluates whether a term without a keyword appears in the same abstract. For “infantile autism disease”, “infantile autism” is extracted and checked if it appears in the same abstract (See the red box in the title).

The first linguistic pattern extracts a candidate phrase by discarding the headword. The candidate phrase is then verified by checking whether the same abstract contains the candidate as a noun phrase without the headword. Figure 1 shows an example of Linguistic Pattern 1. The method first finds the candidate, “infantile autism”, by removing the unique concept headword, “disease”. Next, it searches the abstract whether “infantile autism” is used elsewhere in the same abstract. In this example, “infantile autism” appears in the title, hence it is retained as a candidate phrase. This second step is crucial because it ensures that the candidate is used for a named entity in the abstract.

#### Linguistic Pattern 2

**Figure 2:**
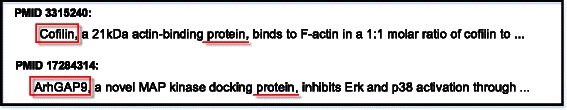
**An example for Linguistic Pattern 2.** This pattern utilizes the pattern, where a term is defined and explained after a “, (appositive)”. “Coflin” and “ArhGAP9” are obtained from the headword, “protein” using this pattern.

The second and the third patterns utilize the written forms, where headwords are used to define other phrases. The second linguistic pattern is, “*X*, a/the... *Y*”, where *X* is a noun phrase and *Y* is a headword. Since *X* is defined by the phrase that includes the headword *Y*, *X* and *Y* may indicate the same concept. Figure 2 presents an example for Linguistic Pattern 2. “Coflin” is defined as “a 21kDa actin-binding protein”. “ArhGAP9” is defined as “a novel MAP kinase docking protein”. Thus, “Coflin” and “ArhGAP9” are reasonable candidates in this example.

#### Linguistic Pattern 3

**Figure 3:**
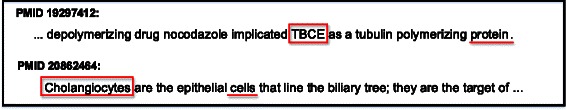
**An example for Linguistic Pattern 3.** This pattern utilizes the pattern, where a term is defined or explained using “is”, “are” or “as”. “TBCE” and “Cholangiocytes” are defined as “a tubulin polymerizing protein” and “the epithelial cells”, respectively.

The last pattern uses the same idea as Linguistic Pattern 2, however it generalizes “is a” relations found in Yeganova et al. [39]. Yeganova et al. proposed an alignment-based method to learn frequent generic patterns that indicate a hyponymy/hypernymy relationship between a pair of noun phrases. Table 2 lists 40 patterns generated by the alignment-based technique. We summarize these patterns as “*X* is/are/as *DT*... *Y*”, where *X* is a noun phrase, *DT* is a determiner and *Y* is a headword. Figure 3 depicts an example for Linguistic Pattern 3. “TBCE” is described as “a tubulin polymerizing protein” and “Cholangiocytes” are described as “the epithelial cells”. Hence, “TBCE” and “Cholangiocytes” become candidate phrases.

**Table 2:** **List of “is a” relations identified in Yeganova et al. [** 39 **]**

X is a Y	X is a potent Y
X are Y	X is the most common Y
X and other Y	X are rare Y
X as a Y	X is a widely used Y
X such as Y	X is an uncommon Y
X is an Y	X is an autosomal dominant Y
X as an Y	X is a form of Y
X is an important Y	X is one of the major Y
X a new Y	X is a chronic Y
X are the most common Y	X and other forms of Y
X is a rare Y	X is a broad spectrum Y
X is a novel Y	X is the primary Y
X is a major Y	X is a rare autosomal recessive Y
X is an essential Y	X is the most common type of Y
X was the only Y	X is the second most common Y
X was the most common Y	X are the most frequent Y
X is a common Y	X is the most widely used Y
X is a new Y	X is the most frequent Y
X is a complex Y	X is the most common primary Y
X is an effective Y	X is one of the major Y

These patterns are summarized as “*X* is/are/as *DT*... *Y*” in our method, where *X* is a phrase, *DT* is a determiner and *Y* is a headword.

The linguistic patterns proposed here are limited to three cases, but they might be extended to include more patterns using automatic knowledge acquisition methods [40,41]. Our study, however, focuses on the overall framework to extract and identify candidate terms from PubMed. An attempt to use automatic knowledge acquisition methods remains as future work.

### Candidate term classification

Candidate phrases obtained from the linguistic patterns may be of good quality already since they are identified from headwords with unique concepts. This is particularly true for Linguistic Patterns 2 and 3. But, Linguistic Pattern 1 may have more noisy terms because it only validates whether candidate phrases are used as named entities. For term extraction, precision is also more important than recall. Therefore, we apply a machine learning classifier to eliminate noisy candidate terms. For this machine learning approach, we first obtain features from training data, i.e. a controlled vocabulary, and an SVM classifier is trained using the extracted features.

#### Features

Our approach uses four feature types: basic, prefix, suffix and headword features. Basic features are trigrams of letters obtained from a phrase [42]. Prefix features add the first two and three characters for each word appearing in a phrase. The headword feature is a special tag for indicating a headword source. Headwords play an important role to identify a concept, hence the features extracted from headwords are handled separately. In addition, suffixes are sometimes a good indicator to decide the concept. For instance, the suffix “-ase” (Table 1) is often used to name enzymes. For this reason, suffix features add the last three, four and five letters from a headword if the headword contains more than five characters.

Here are the features obtained from the phrase, “mosaic virus”:

- Prefix features (“$”): “mo$”, “mos$”
- Common features: “mos”, “osa”, “sai”, “aic”
- Prefix features (“$”) from the headword (“!h”) : “vi$!h”, “vir$!h”
- Common features from the headword (“!h”): “vir!h”, “iru!h”, “rus!h”

There are no suffix features in this example because the headword, “virus”, has only 5 characters.

#### SVM classifiers

We apply an SVM classifier with the modified Huber loss function [43,44] for filtering noisy terms that are introduced by the linguistic patterns. This method follows standard SVM learning, but replaces the hinge loss function with the modified Huber loss function [43].

Let *T* denote the size of the training set and $\overrightarrow {X_{i}}$ be the binary feature vector of the *i*th example in the training set. The class indicator *y*_*i*_=1 if the example is annotated as positive and *y*_*i*_=−1 otherwise. Let $\overrightarrow {w}$ denote a vector of feature weights for $\overrightarrow {X_{i}}$. Let *θ* denote a threshold parameter, and let *λ* denote a regularization parameter. The cost function is then given by

(1) $$\begin{array}{@{}rcl@{}} C = \frac{1}{2} \lambda |\overrightarrow{w}|^{2} + \frac{1}{T} \sum_{i=1}^{T} h \left(y_{i} \left(\theta + \overrightarrow{w} \cdot \overrightarrow{X_{i}}\right)\right), \end{array} $$

where the function *h* is the modified Huber loss function,

(2) $$\begin{array}{@{}rcl@{}} h(z) = \left\{ \begin{array}{ll} -4z, &\text{if}\ z \leq -1, \\ (1-z)^{2}, &\text{if}\ -1<z<1, \\ 0, &\text{if}\ 1 \leq z. \end{array} \right. \end{array} $$

The values of $\overrightarrow {w}$ and *θ* minimizing *C* are determined using a gradient descent algorithm. The regularization parameter *λ* is computed from the training set as

(3) $$\begin{array}{@{}rcl@{}} \lambda = \lambda' \langle|\overrightarrow{x}|\rangle^{2}, \end{array} $$

where $\langle |\overrightarrow {x}|\rangle $ is the average Euclidean norm of the feature vectors in the training set. For experiments, the parameter *λ* ^′^ was adjusted to maximize 10-fold cross-validation performance on the training set, which yielded 0.0000001. The modified Huber function was used in our approach as it has produced good performance in other classification problems [31,45]. However, there was no significant improvement compared to using the hinge loss function in the proposed term extraction problem.

## Results and discussion

### Dataset

The proposed method requires a training set for the SVM classifier. For training, we need a controlled vocabulary for “gene”, “protein”, “disease” and “cell(s)”, and the SemCat [31] database is used for creating positive and negative sets. SemCat is not publicly available due to license issues for some resources included.

Table 3 presents the number of positive and negative examples employed for each headword. For positive sets, the SemCat categories, “GENE_OR_PROTEIN/DNA_MOLECULE”, “GENE_OR_PROTEIN/PROTEIN_MOLECULE”, “DISEASE_OR_SYNDROME/INJURY_OR_POISONING/SIGN_OR_SYMPTOM” and “CELL” are used for the headwords, “gene”, “protein”, “disease” and “cell(s)”, respectively. For a given category, all terms from other SemCat categories are utilized for negative sets. Moreover, all terms are lemmatized [46], and only PubMed-compatible terms are included to remove the terms not compatible with PubMed as well as to reduce the size of training sets. A SemCat term is called compatible if all contiguous bigrams appearing in the SemCat term also appear in PubMed abstracts. For candidate term extraction, PubMed abstracts (July, 2014) are used, and this collection contains more than 24 million records.

**Table 3:** **Dataset used for training SVM classifiers**

**Headwords**	**Positive**	**Negative**	**SemCat catogories**
Gene	3532163	1631676	GENE_OR_PROTEIN
			DNA_MOLECULE
Protein	3533621	1630690	GENE_OR_PROTEIN
			PROTEIN_MOLECULE
Disease	88653	5096888	DISEASE_OR_SYNDROME
			INJURY_OR_POISONING
			SIGN_OR_SYMPTOM
Cell(s)	14581	5178142	CELL

For each keyword, terms from relevant SemCat categories were merged and used for the classifiers.

### Noun phrase detection

In our experiments, MedPost [47] was used for Part-Of-Speech (POS) tagging, and all programs were implemented in C/C++. To identify a noun phrase for a given headword, the headword (noun) is first found. If there is another noun to the right, the phrase is not considered as a correct noun phrase for extraction. Starting from the headword found in a sentence, add tokens adjacent to the left successively as long as they are adjectives, nouns or numbers. The result is a noun phrase of interest for further processing.

### SVM performance

The SVM classifier is applied in the last step, and filters candidate phrases to include only correct terms as output. Therefore, it is important to have high precision performance in this stage. Table 4 shows precision, recall and F1 scores for “gene”, “protein”, “disease” and “cell(s)” using 10-fold cross-validation on the training set. In the table, both precisions and recalls are higher than 97% for the headwords, “gene” and “protein”. However, recalls drop to 75.55% and 66.94% for “disease” and “cell(s)”, respectively. This is due to the imbalance between the number of positive and negative examples. Nevertheless, precisions are still high, producing 94.08% on average. Thus, we expect highly accurate terms after the SVM classification even though we will lose some candidate phrases.

**Table 4:** **SVM performance using 10-fold cross-validation on the training set for five keywords, “gene”, “protein”, “disease” and “cell(s)”**

**Headwords**	**Precision**	**Recall**	**F1**
Gene	0.9721	0.9838	0.9779
Protein	0.9738	0.9846	0.9792
Disease	0.8938	0.7555	0.8188
Cell(s)	0.9233	0.6694	0.7761

In our study, the modified Huber loss function was used for the SVM classifier, but the performance improvement was not significant over the standard SVM using the hinge loss function. The SVM classifier with the modified Huber loss function produced 97.79%, 97.92%, 81.88% and 77.61% F1 for “gene”, “protein”, “disease” and “cell(s)”, respectively (Table 4), whereas the SVM classifier with the hinge loss function showed 97.47%, 97.41%, 81.94% and 76.75% F1 for the same sets.

### Performance on term extraction

After applying the linguistic patterns to PubMed abstracts, and obtaining candidate terms, followed by applying the SVM classifier to these terms, we obtained a total of 88,384 unique phrases. Among these phrases, 57,614 terms were already in SemCat. 30,770 terms (35%) were new, i.e. did not exist in current SemCat. Three reviewers evaluated new terms, but for the headwords, “gene”, “protein” (All Linguistic Patterns) and “disease” (Linguistic Pattern 3), there were many newly discovered terms. In these cases, 100 terms were randomly selected and used for evaluation. See “Additional file 1” for all the terms used for evaluation and their annotation results.

Tables 5, 6 and 7 present the size of evaluated sets and precisions for the terms evaluated by three reviewers. “gene” and “protein” are dominant in terms of the number of extracted entities as PubMed is a major resource covering molecular biology, and genes and proteins outnumber diseases and cell types by a wide margin. As shown in the tables, all the three linguistic patterns achieve over 90% precision for the headwords, “gene”, “protein”, “disease”, “cell” and “cells”. Overall, Linguistic Patterns 2 and 3 produce more accurate results than Linguistic Pattern 1. This is because Linguistic Patterns 2 and 3 find terms that are defined by the headwords using “, (appositive)” or “is/are/as”, whereas Linguistic Pattern 1 encounters more general terms.

**Table 5:** **Performance for Linguistic Pattern 1**

**Headwords**	**Total**	**New**	**Evaluated**	**Reviewer 1**	**Reviewer 2**	**Reviewer 3**
Gene	37678	12461	100	91.0%	91.0%	91.0%
Protein	24000	8630	100	91.0%	91.0%	91.0%
Disease	438	163	163	93.9%	94.5%	93.3%
Cell	50	21	21	95.2%	95.2%	95.2%
Cells	565	380	380	97.1%	97.6%	97.4%

Precisions for each annotator are shown for “gene”, “protein”, “disease”, “cell” and “cells”. “Total” means the total number of obtained terms. “New” and “Evaluated” mean the number of terms not in SemCat and the number of evaluated terms by reviewers, respectively.

**Table 6:** **Performance for Linguistic Pattern 2**

**Headwords**	**Total**	**New**	**Evaluated**	**Reviewer 1**	**Reviewer 2**	**Reviewer 3**
Gene	1285	386	100	77.0%	77.0%	76.0%
Protein	3484	1048	100	93.0%	93.0%	93.0%
Disease	274	64	64	98.4%	98.4%	96.9%
Cell	77	63	63	98.4%	98.4%	98.4%
Cells	56	30	30	96.7%	96.7%	96.7%

Precisions for each annotator are shown for “gene”, “protein”, “disease”, “cell” and “cells”. “Total” means the total number of obtained terms. “New” and “Evaluated” mean the number of terms not in SemCat and the number of evaluated terms by reviewers, respectively.

**Table 7:** **Performance for Linguistic Pattern 3**

**Headwords**	**Total**	**New**	**Evaluated**	**Reviewer 1**	**Reviewer 2**	**Reviewer 3**
Gene	5098	1230	100	90.0%	90.0%	90.0%
Protein	10439	3847	100	91.0%	91.0%	91.0%
Disease	4681	2298	100	99.0%	99.0%	99.0%
Cell	147	80	80	95.0%	95.0%	95.0%
Cells	112	69	69	98.6%	98.6%	98.6%

Precisions for each annotator are shown for “gene”, “protein”, “disease”, “cell” and “cells”. “Total” means the total number of obtained terms. “New” and “Evaluated” mean the number of terms not in SemCat and the number of evaluated terms by reviewers, respectively.

In our study, it is impossible to evaluate recall because true labels are not available for PubMed terms. However, a useful estimation is possible by calculating recalls based on SemCat terms. Table 8 shows the estimated recalls for PubMed in Linguistic Patterns 1, 2 and 3. Recalls were evaluated based on number of SemCat terms occurring in PubMed that were discovered by the pattern. The results are 14.0%, 10.6%, 4.8%, 2.4% and 2.8% recall overall for “gene”, “protein”, “disease”, “cell” and “cells”, respectively. Linguistic Patterns 1 and 3 describe more general forms for term extraction, hence these patterns yield higher recall than Linguistic Pattern 2. Table 9 presents the estimated recalls for PubMed without applying SVM classifiers. The recalls increase overall without SVM classification, discovering 16.3% of terms on average. Note that the main goal of the proposed framework is to extract new terms that do not appear in a standard terminology. Incorporating more patterns [40,41] would increase the recall.

**Table 8:** **Estimated recalls for Linguistic Patterns 1, 2 and 3**

**Headwords**	**Pattern 1**	**Pattern 2**	**Pattern 3**	**Total**
Gene	13.5%	0.6%	2.4%	14.0%
Protein	8.7%	1.6%	3.9%	10.6%
Disease	0.5%	0.4%	4.5%	4.8%
Cell	0.7%	0.5%	2.1%	2.4%
Cells	1.8%	0.9%	1.3%	2.8%
Average	5.0%	0.8%	2.8%	6.9%

As no true labels are available for PubMed terms, recalls were evaluated based on number of SemCat terms occurring in PubMed that were discovered by the pattern.

**Table 9:** **Estimated recalls for Linguistic Patterns 1, 2 and 3 without SVM classification**

**Headwords**	**Pattern 1**	**Pattern 2**	**Pattern 3**	**Total**
Gene	17.4%	0.8%	3.1%	18.2%
Protein	11.6%	2.3%	5.4%	14.2%
Disease	1.4%	0.6%	6.1%	6.8%
Cell	8.0%	1.0%	3.5%	10.7%
Cells	29.7%	1.7%	2.7%	31.6%
Average	13.6%	1.3%	4.2%	16.3%

As no true labels are available for PubMed terms, recalls were evaluated based on number of SemCat terms occurring in PubMed that were discovered by the pattern.

Although our approach shows high precision overall, the headword “gene” in Linguistic Pattern 2 (Table 6) provides only 77% precision. There are two common errors that lead to incorrectly predicted terms. Here are a few examples from the first type of error.

- They harbour a class 1 integron with an aadA1 gene in the 855 ***bp variable region***, a tet(A) ***gene***,...
- we introduced an oncogenic component of ***HBV***, the hepatitis B virus X (HBx) ***gene***,...
- Each repeat consists of the 35S rRNA gene, the ***NTS1 spacer***, the 5S rRNA ***gene***, and the NTS2 spacer.

As shown in the above, these errors occur because we ignore some of the syntactic structures (phrase attachment). Currently, we simply match the proposed linguistic patterns without attention to such detail. Enumerations could be recognized and excluded at some level of accuracy. The errors coming from incorrect parsing are more complicated. Improvement here may come by either taking account of detailed syntactic analysis or improving SVM classifier performance. Unlike the previous case, the second error type is caused by semantics. The following are some examples.

- ... were processed and sectioned to localize ***histone 3 mRNA***, a cell cycle specific ***gene***, by in situ hybridization.
- Expression of ***megsin mRNA***, a novel mesangium-predominant ***gene***, in the renal tissues of various glomerular diseases.

During the review process, we decided to exclude terms that were clearly considered as different concepts. The examples here define “histone 3 mRNA” and “megsin mRNA” as genes. But, there is the category, “RNA_MOLECULE” in SemCat. It is our convention that mRNA terms belong to “RNA_MOLECULE”. “mRNA” is the only semantic case we found from our analysis. This can be dealt with by building a rule, e.g. {mRNA} → {RNA_MOLECULE}.

Another case we found from error analysis is that a term is valid, but the meaning is too general. For instance, “fourth cell type” and “single cell type” clearly indicate a type of cells, but it is uncertain what the cell type means. Such terms are not useful for enriching SemCat. Thus, we manually re-evaluated all the candidate terms using a modified guideline, i.e. a term is marked as incorrect if the term is too general. Table 10 shows precision results with and without considering general terms. Precisions do not change much for most cases, but there is a relatively big impact on the headword “cell” for Linguistic Patterns 2 and 3. This may be a unique feature of how the headword “cell” is mentioned in articles. A simple solution for this problem is to adopt a stopword list for eliminating general terms, but a careful design is necessary to build and apply stopwords for candidate terms.

**Table 10:** **Performance comparison with or without including general terms**

**Headword**	**Original evaluation**			**General terms excluded**		
	**Pattern 1**	**Pattern 2**	**Pattern 3**	**Pattern 1**	**Pattern 2**	**Pattern 3**
Gene	91.0%	76.7%	90.0%	90.0%	73.0%	88.0%
Protein	91.0%	93.0%	91.0%	90.0%	89.0%	85.0%
Disease	93.9%	97.9%	99.0%	93.9%	96.4%	99.0%
Cell	95.2%	98.4%	95.0%	95.2%	71.4%	88.8%
Cells	97.4%	96.7%	98.6%	96.4%	90.0%	95.7%

“General” indicates a term is valid, but the meaning is too general and not useful for enriching SemCat. Scores are the precisions averaged from three reviewers.

## Conclusions

Most of the term identification methods currently available focus on detecting one or a few entities, hence dictionary- or hybrid-based approaches have more advantages in this regard. However, the low overlap between standard terminologies and terms in biomedical literature is a major difficulty to widely adopt controlled vocabularies for term identification. In this paper, we seek a solution by enriching semantic categories using entities in PubMed. The proposed method first finds headwords identified with unique concepts, and linguistic patterns are applied to extract candidate terms related to the headwords. Finally, an SVM classifier is utilized for removing incorrect terms. For experiments, the SVM classifier was trained on SemCat terms, and candidate terms were obtained from PubMed abstracts. The experimental results demonstrate that the proposed method is promising by achieving 93% precision on average for the headwords, “gene”, “protein”, “disease”, “cell” and “cells”.

Although our approach shows good performance, our analysis suggests that more work needs to be done. Errors occur in three different forms: 1) enumeration or syntactic error, 2) semantic error and 3) general term error. As future work, we plan to address the first and the third error types. More precise Part-Of-Speech (POS) tagging and syntactic parsing can decrease enumeration and syntactic errors. General terms may be reduced by developing a stopword list for our extraction technique.

## Additional file

Additional file 1
**Terms evaluated and annotation results.** The terms used for manual evaluation are presented for “gene”, “protein”, “disease”, “cell” and “cells”. Annotated results for three reviewers are also listed together.
